# ncDLRES: a novel method for non-coding RNAs family prediction based on dynamic LSTM and ResNet

**DOI:** 10.1186/s12859-021-04365-4

**Published:** 2021-09-20

**Authors:** Linyu Wang, Xiaodan Zhong, Shuo Wang, Yuanning Liu

**Affiliations:** College of Computer Science and Technology, Jilin University, Changchun, China

**Keywords:** ncRNAs family, ResNet, LSTM, ncDLRES

## Abstract

**Background:**

Studies have proven that the same family of non-coding RNAs (ncRNAs) have similar functions, so predicting the ncRNAs family is helpful to the research of ncRNAs functions. The existing calculation methods mainly fall into two categories: the first type is to predict ncRNAs family by learning the features of sequence or secondary structure, and the other type is to predict ncRNAs family by the alignment among homologs sequences. In the first type, some methods predict ncRNAs family by learning predicted secondary structure features. The inaccuracy of predicted secondary structure may cause the low accuracy of those methods. Different from that, ncRFP directly learning the features of ncRNA sequences to predict ncRNAs family. Although ncRFP simplifies the prediction process and improves the performance, there is room for improvement in ncRFP performance due to the incomplete features of its input data. In the secondary type, the homologous sequence alignment method can achieve the highest performance at present. However, due to the need for consensus secondary structure annotation of ncRNA sequences, and the helplessness for modeling pseudoknots, the use of the method is limited.

**Results:**

In this paper, a novel method “ncDLRES”, which according to learning the sequence features, is proposed to predict the family of ncRNAs based on Dynamic LSTM (Long Short-term Memory) and ResNet (Residual Neural Network).

**Conclusions:**

ncDLRES extracts the features of ncRNA sequences based on Dynamic LSTM and then classifies them by ResNet. Compared with the homologous sequence alignment method, ncDLRES reduces the data requirement and expands the application scope. By comparing with the first type of methods, the performance of ncDLRES is greatly improved.

## Background

RNA is a kind of biological macromolecule composed of nucleotides, which mainly contains four kinds of nucleotide: A (Adenine), U (Uracil), G (Guanine), and C (Cytosine) [1]. There are many ways for RNA to participate in life activities. According to its different ways, RNA can be classified into two types: coding RNAs and non-coding RNAs. To be specific, coding RNAs are translated into protein by translation rules. Since protein undertakes most of the life activities, coding RNAs have been the focus of research for decades, while non-coding RNAs (ncRNAs) are functional RNAs that are transcribed from ncRNA genes but do not encode proteins [2], which play important roles in various cellular processes [3] and diseases [4] by means of replication, transcription, or gene expression regulation [5, 6]. According to transcriptomic and bioinformatics studies, there are thousands of ncRNAs classified into different categories based on their functions and lengths [2] including microRNA, rRNA, ribozymes, snRNA (snoRNA), tRNA, Intron_RNA, IRES, leader, and riboswitch. microRNA is $\sim$22 nt RNA molecule, which can affect protein expression by targeting other molecules and then regulate life activity [7]. rRNA is the basic material of life, which is involved in protein transcription and plays a regulatory role in the cell [8]. ribozymes is a kind of RNA enzyme in the organism, which plays the role of connecting amino acids in protein synthesis [9]. snRNA (snoRNA) refers to a class of RNA molecules with a length of $\sim$150 nt, and its main function consists of processing the pre-messenger RNA (hnRNA) in the nucleus, regulating transcription factors, and maintaining telomeres [8]. tRNA is a class of RNA molecules with a length of about 76–90 nt, which can act as a physical link between mRNA and amino acid sequences [10]. As for Intron_RNA, it is a kind of RNA that is transcribed from the intron gene. After being transcribed into RNA, they carry out extensive internal interaction and help exons to join together in the right order [11]. IRES can assist in ribosome binding with messenger RNA to initiate protein translation and synthesis [12]. In terms of the leader, it is the upstream segment of the start codon in mRNA, and plays an important role in regulating the transcription of mRNA [13]. riboswitch is a regulatory fragment of mRNA, which can regulate the process of mRNA transcription by folding into a certain conformation [14].

The emergence of high-throughput technology reduces the time and labor cost of gene sequencing to a great extent [15]. Researchers have discovered a large number of unknown ncRNA sequences by adopting high-throughput technology. The functional research of these sequences has brought great pressure to biologists, and studies have shown that the same family of ncRNAs have similar functions, and thus, the identification of ncRNAs family can preliminarily determine their function, and then promote the functional research of ncRNAs. It is time-consuming and laborious to identify ncRNAs family by biological experiments, which cannot meet the needs of high-throughput data. Therefore, computational methods are required to quickly realize ncRNAs family recognition. The existing methods for predicting ncRNAs family can be divided into two categories: the first type is to predict ncRNAs family by learning the features of sequence or secondary structure, and the other type is to predict ncRNAs family by the alignment among homologs sequences. In the first type, some methods (GraPPLE [16], RNAcon [17], and nRC [18]) predict ncRNAs family by learning predicted secondary structure features. In these methods, various RNA secondary structure prediction tools [19, 20] are used to obtain ncRNAs secondary structure, and then design calculation methods based on predicted secondary structure features to classify ncRNAs. At present, the performance of secondary structure prediction tools is not perfect, which leads to large errors of secondary structure prediction. These methods use predicted structural features with large errors to classify ncRNAs will make the performance is low. The other is prediction method “ncRFP” [21] proposed by our team. In this method, deep learning is employed to directly extract the features of ncRNA sequences and classify ncRNAs. Compared with those methods based on the features of secondary structure, ncRFP simplifies the prediction process, reduces the errors, and improves the prediction efficiency. Due to the static deep learning model adopted in ncRFP, ncRNA sequences should be preprocessed into the same length. During the data preprocessing, ncRNA sequences of different lengths will be padded/truncated to the same length sequences, which results in the loss of the features. Hence, there is room for improvement in ncRFP performance. In the secondary type, Infernal [22] is the representative of the homologous sequence alignment method, which based on the structurally annotated multiple sequence alignment to identify ncRNAs family. Rfam [23] is a ncRNAs database of multiple families, which contains not only the ncRNA sequences, but also the aligned sequences with consensus secondary structure annotation. Therefore, Infernal can adopt the structural annotation data in Rfam to create covariance models (CMs) based on the stochastic context-free grammars (SCFGs) [24]. Then Infernal use those CMs to accurately identify ncRNAs family. In some families with complete secondary structure annotation data, the accuracy of Infernal is very high, which makes Infernal widely used. Although Infernal can achieve high performance, it still has some defects. Due to the need for consensus secondary structure annotation data, when some families only have sequence data or inaccurate secondary structure annotation data, the performance of Infernal will be low in those families due to the lack of necessary data. At the same time, the pseudoknots in RNA secondary structure can not be modeled by Infernal, which will reduce the accuracy of some families with pseudoknots. Based on the advantages and disadvantages of those existing methods, it is necessary to propose a novel method to predict ncRNAs family. The new method not only needs to improve the performance compared with those methods based on the features of sequence or secondary structure, but also reduces the demand for data to expand the application scope compared with Infernal.

## Results

In this paper, a novel method “ncDLRES” is proposed to predict ncRNAs family based on a dynamic deep learning model. ncRNAs have a three-hierarchy structure: primary structure, secondary structure, and tertiary structure, which are corresponding to ncRNAs sequence, two-dimensional plane structure, and three-dimensional spatial structure respectively. Each hierarchy structure of ncRNAs contains family characteristics, which can be used as the input of the deep learning model. Because of the primary structure obtained accurately according to the high-throughput technology, ncDLRES adopts ncRNA sequences as input data to classify ncRNAs according to their primary structural features, which can effectively extract the most accurate family features and improve the prediction performance. In the static deep learning model, the input data should be padded or truncated into the same format, which will increase noise or loss features. Hence, ncDLRES adopts a dynamic deep learning model, which can take ncRNA sequences of different lengths as input data and preserve the complete features of ncRNA sequences. ncDLRES includes Dynamic LSTM [25] and ResNet [26]. As for Dynamic LSTM, it is responsible for encoding ncRNAs of different lengths into the same format data, while the ResNet tends to classify the encoded data. In order to improve the performance, ncDLRES also employs the Attention Mechanism [27] to focus algorithm attention on important segments. Compared with the method by learning to the secondary structure features, ncDLRES simplifies the prediction process, while different from ncRFP, this method preserves the integrity of input data. Compared with the homologous sequence alignment method, ncDLRES only needs the primary structure to identity ncRNAs family, reduces the data requirement and expands the application scope.

### Learning results and presentation

**Fig. 1:**
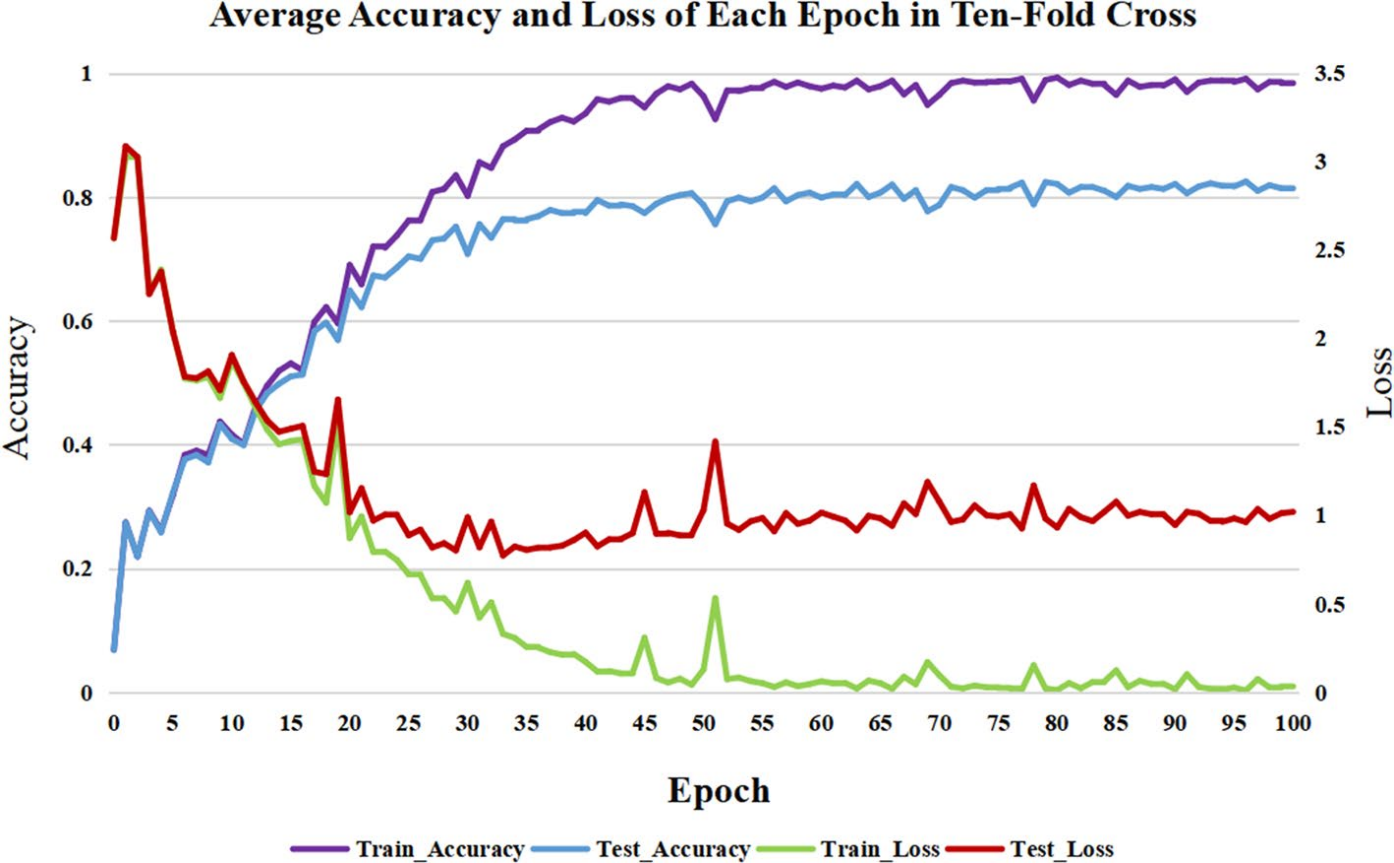
Average accuracy and loss of train and test sets in cross data. The violet curve, blue curve, red curve and green curve indicate training accuracy, testing accuracy, testing loss and training loss respectively

In the model learning process, all of the ncRNAs data is processed into ten-fold cross-validation train and test sets, and ncDLRES is trained and tested 100 epochs in each fold of train and test sets. Figure 1 is the average accuracy and loss of ten-fold cross-validation in each epoch of training and testing.The loss is calculated by the cross-entropy loss function (Eq. 1). It can be seen from the figure that although the curve fluctuates, which may be caused by the higher learning rate, there is no phenomenon of over-fitting or under-fitting, and the accuracy and loss of the test set are stable in the final epochs, which shows that the model can be competent for the task of ncRNAs family prediction.

1 $$\begin{aligned} L (y, f (x)) = -\frac{1}{N}\sum _{i=1}^N log[q ({\hat{y}}_i = c_k|x_i)] .\end{aligned}$$

### Prediction results and comparison

In this section, the prediction results of ncDLRES will be presented and compared with GraPPLE [16], RNAcon [17], nRC [18], and ncRFP [21]. In this paper, the performance of ncDLRES is compared with those four methods in two aspects. The first aspect is to compare the average performance of the ten-fold test data, while the other aspect is to compare the average performance of the single-family in the ten-fold test data. In order to make the performance evaluation more perfect, Accuracy, Sensitivity, Precision, F-score, and MCC are employed to evaluate the performance of multi-methods. Accuracy is the ratio of all the correct prediction ncRNA sequences to all the ncRNA sequences; Sensitivity is the proportion of the correct prediction data in one whole family data; Precision is the proportion of the number of the correct prediction in the whole predicted number of one family; F-score is the weighted harmonic mean of Sensitivity and Precision, and MCC is an index used to measure the classification performance. Their formulas are as follows (Eqs. 2–6), where TP, TN, FP, and FN are True Positives, True Negative, False Positives, and False Negatives respectively.

2 $$\begin{aligned} Accuracy = \frac{TP + TN}{TP + TN + FP + FN} \end{aligned}$$

3 $$\begin{aligned} Sensitivity = \frac{TP}{TP + FN} \end{aligned}$$

4 $$\begin{aligned} Precision = \frac{TP}{TP + FP} \end{aligned}$$

5 $$\begin{aligned} F\text {-}score = \frac{2*TP}{2*TP + FP + FN} \end{aligned}$$

6 $$\begin{aligned} MCC = \frac{TP*TN-FP*FN}{\sqrt{ (TP+FP)* (TP+FN)* (TN +FP)* (TN+FN)}} .\end{aligned}$$

**Fig. 2:**
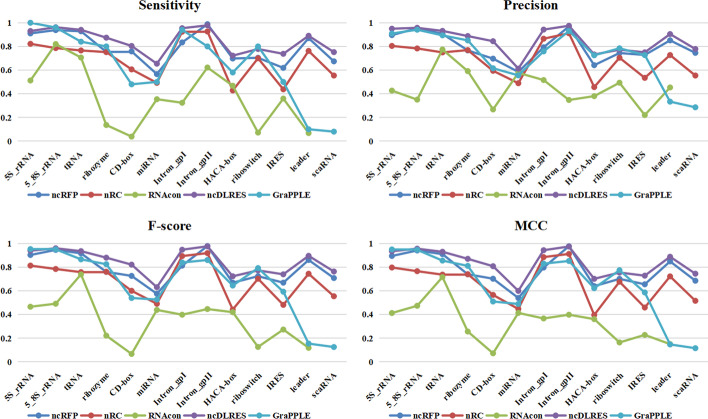
Performance comparison of different families. The violet curve, dark blue curve, red curve, green curve, and baby blue curve represent the performance of ncDLRES, ncRFP, nRC, RNAcon, and GraPPLE respectively

Table 1 shows the average performance of ten-fold test sets in multiple methods. It can be seen from the table that ncDLRES is superior to other methods in all indexes and reaches the optimal level. Figure 2 shows the comparison of the single-family in different indexes. It can be seen from the figure that ncDLRES is optimal in almost all families, only in Intron_GpII, it is slightly lower than ncRFP, and in 5S_rRNA and riboswitch, it is lower than GraPPLE.

**Table 1:** Performance comparison between multiple methods

Method	Accuracy	Sensitivity	Precision	F-score	MCC
RNAcon	0.3737	0.3732	0.4497	0.3505	0.3341
nRC	0.6960	0.6889	0.6878	0.6878	0.6627
GraPPLE	0.6487	0.6480	0.7721	0.7050	0.6857
ncRFP	0.7972	0.7878	0.7904	0.7883	0.7714
ncDLRES	**0.8479**	**0.8448**	**0.8489**	**0.8451**	**0.8335**

The bold value is the maximum of each column

## Discussion

RNAs are important biological macromolecules, which can participate in the regulation of life activities in a variety of ways. They can be mainly divided into two types, coding RNAs and non-coding RNAs (ncRNAs). Coding RNAs regulate life activities by translating into proteins. Since proteins undertake a variety of life tasks, coding RNAs can be studied by researching the function of proteins. In recent years, with the in-depth research on ncRNAs, an increasing amount of evidence has shown that ncRNAs involved in a variety of life regulation activities. Therefore, studying the function of ncRNAs is beneficial to the research of life science. Studies have shown that the same family of ncRNAs is featured with similar functions. Therefore, their function can be preliminarily determined by predicting the ncRNAs family. In the high-throughput era, time-consuming and laborious biological experimental methods cannot meet the needs of scientific research. In this case, computational methods are necessary to predict ncRNAs family. Since it is difficult to obtain accurate secondary structure, the performance of those methods based on secondary structure features is low. Although the performance of ncRFP, which based on the primary structure features, is better than those methods based on secondary structure, it cannot achieve the best performance due to the loss of input features. Infernal can achieve very high accuracy when it has consensus secondary structure annotation data. However, due to its high requirements for data, its application scope is limited. At the same time, Internal cannot model pseudoknots in the secondary structure, which will reduce the accuracy of some families with pseudoknots. Therefore, it is necessary to propose a new method to avoid those defects of the existing methods. In this paper, a novel method “ncDLRES” is proposed to predict the family of ncRNAs based on a dynamic deep learning model. Its input is ncRNA sequences, whose features are more accurate than those methods based on secondary structure and ncDLRES uses a dynamic deep learning model to avoid the loss of input features compared with ncRFP. Furthermore, ncDLRES only needs ncRNA sequences to predict ncRNAs family, which reduces the demand for data compared with Infernal. Hence, it not only can be applied to families with consensus secondary structure annotation data, but also can be applied to families with only sequence data, inaccurate structure annotation data, or pseudoknots data, which expands the scope of application and avoids the defect of cannot model pseudoknots.

**Fig. 3:**
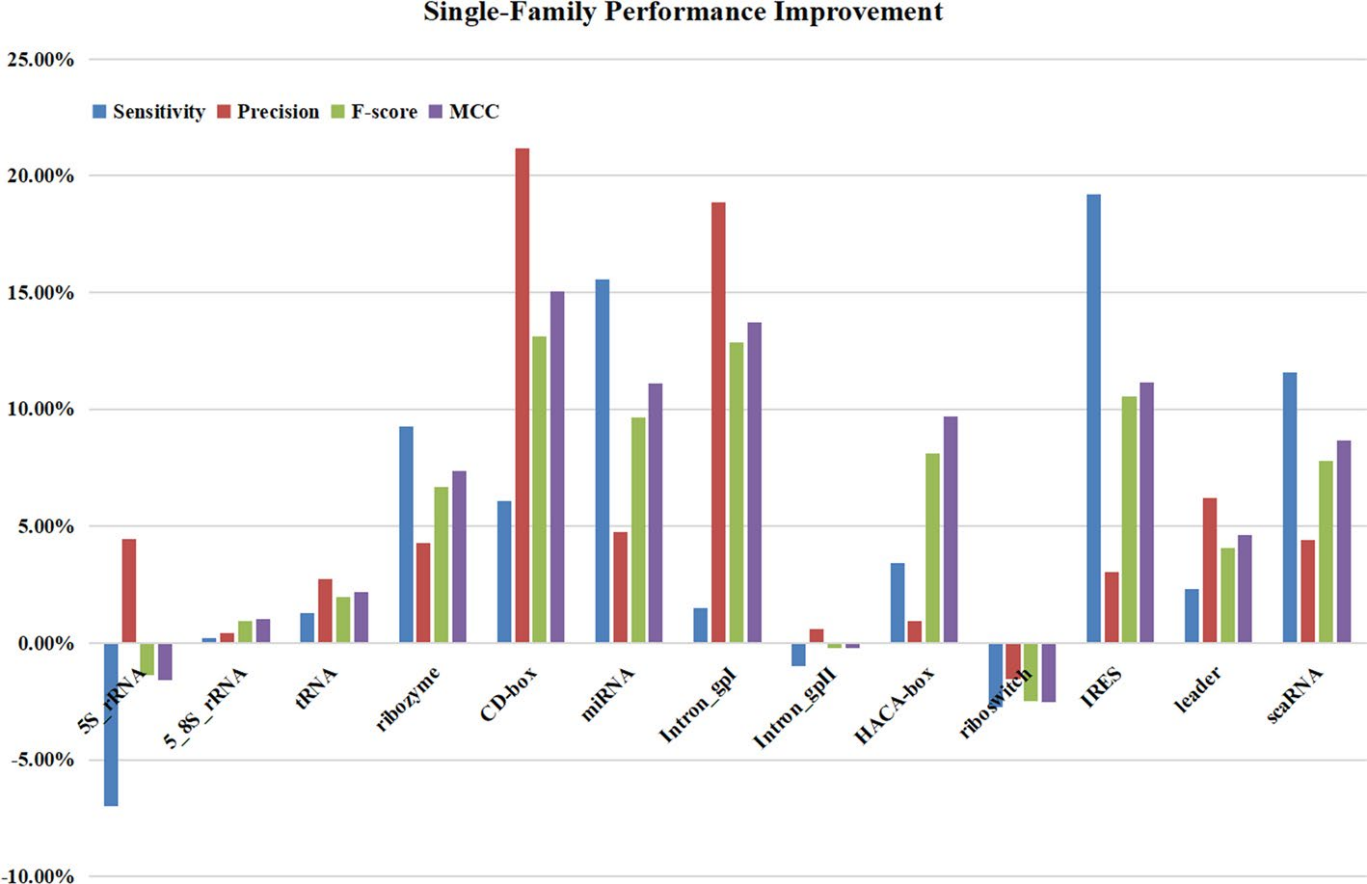
Single-family performance improvement. The violet column, blue column, red column and green column represent Sensitivity, Precision, F-score, and MCC respectively

In this paper, the performance of ncDLRES is compared with that of several excellent methods including ncRFP based on primary structure features, and GraPPLE RNAcon and nRC based on secondary structure features. ncRFP adopts a static deep learning model to directly learning ncRNAs sequence features to classify ncRNAs. GraPPLE adopts SVM to classify the ncRNAs based on the graph properties of the predicted secondary structure. RNAcon extracts 20 graph features from the predicted secondary structure, and then designs random forest to classify ncRNAs based on the extracted features. nRC adopts the Moss [28] to extract and encode the features of predicted secondary structure, and then designs a convolutional neural network to classify ncRNAs. The experimental conditions, which only need ncRNA sequences to predict ncRNAs family, of those four methods are the same as ncDLRES. At the same time, these four methods are excellent in the prediction methods based on sequence or secondary structure features. Therefore, those four methods are chosen to compare with ncDLRES. The comparison is made from the whole data and the single-family data. Table 1 shows the average performance of different methods in the whole data. Then, it is found that the performance of ncDLRES is optimal among all indexes. Accuracy, Sensitivity, Precision, F-score, and, MCC are improved by 6.35%, 7.23%, 7.4%, 7.2%, and 8.05% respectively compared with the suboptimal ncRFP. Figure 2 shows the comparison concerning the average performance of the single-family. As precision has the same meaning as accuracy in the single-family, accuracy is not included in the single-family performance comparison. The diagram shows that ncDLRES in microRNAs, 5.8S_rRNA, ribozymes, CD-BOX, HACA-BOX, scaRNA, tRNA, Intron_GPI, IRES, and leaders achieves the optimal performance, only in Intron_GpII, the performance is slightly lower than ncRFP,and in 5S_rRNA and riboswitch, the performance is lower than GraPPLE. Figure 3 shows the details of the increase and decrease of different indexes in single-family compared between the optimal method ncDLRES and the suboptimal methods ncRFP and GraPPLE. The reason why ncDLRES achieves the best performance in the whole data and most single-family is that it uses dynamic deep learning model can use complete ncRNA sequence as input, which makes the extraction of primary structure features more comprehensive and accurate. On the family of Inron_GpII, the performance of ncDLRES is slightly lower than that of ncRFP, maybe because that the Inron_GpII family features are more significant than other families in the process of data padding and truncation. On the family of 5S_rRNA and riboswitch, the performance of ncDLRES is lower than that of GraPPLE, maybe because that the secondary structure graph features of those two families are remarkable, which makes GraPPLE more suitable to identify them.

## Conclusions

In the performance comparison, the performance of ncDLRES has been greatly improved, which means that ncRNAs family prediction can be completed based on the sequence characteristics. Compared with the existing methods, ncDLRES has many advantages. Firstly, the prediction process of ncDLRES does not involve RNA secondary structure, which not only simplifies the prediction process, but also reduces the loss caused by multi-step error superposition compared with the method based on secondary structure feature. Meanwhile, compared with Infernal, it can avoid the defect of inferior performance caused by the lack of structure annotation data or the pseudoknots data. Secondly, ncDLRES uses the dynamic deep learning model to process ncRNA sequences of various lengths, which can avoid feature loss and improve performance. Finally, in the field of application, it can not only complete the prediction alone, but also complete the prediction in cooperation with Infernal. ncDLRES can further improve the performance reliability of Infernal by predicting the families with good performance in Infernal. In the families with the lack of structure annotation data or the pseudoknots data, which are not good at by Infernal, ncDLRES can make up for the deficiency of Infernal by learning sequence features, so that those two methods can be combined to better serve the family prediction of ncRNAs. Although there are many advantages of ncDLRES, it still has some defects that need to be solved in the future. The performance of ncDLRES is not optimal among the families of Inron_GpII, 5S_rRNA, and riboswitch, which has room for improvement. The performance of ncDLRES in Inron_GpII is lower than that of ncRFP, which indicates that the static model still has some advantages that should learn. The performance of ncDLRES in 5S_rRNA and riboswitch is lower than that of GraPPLE, which indicates that the secondary structure features can also recognize some families with high accuracy. In the future, we will combine the advantages of ncDLRES, ncRFP, and GraPPLE to create a new ncRNAs recognition method with better performance, and establish ncRNAs family recognition website to provide services for researchers and contribute to life science research.

## Materials and method

### Data collection and processing

**Fig. 4:**
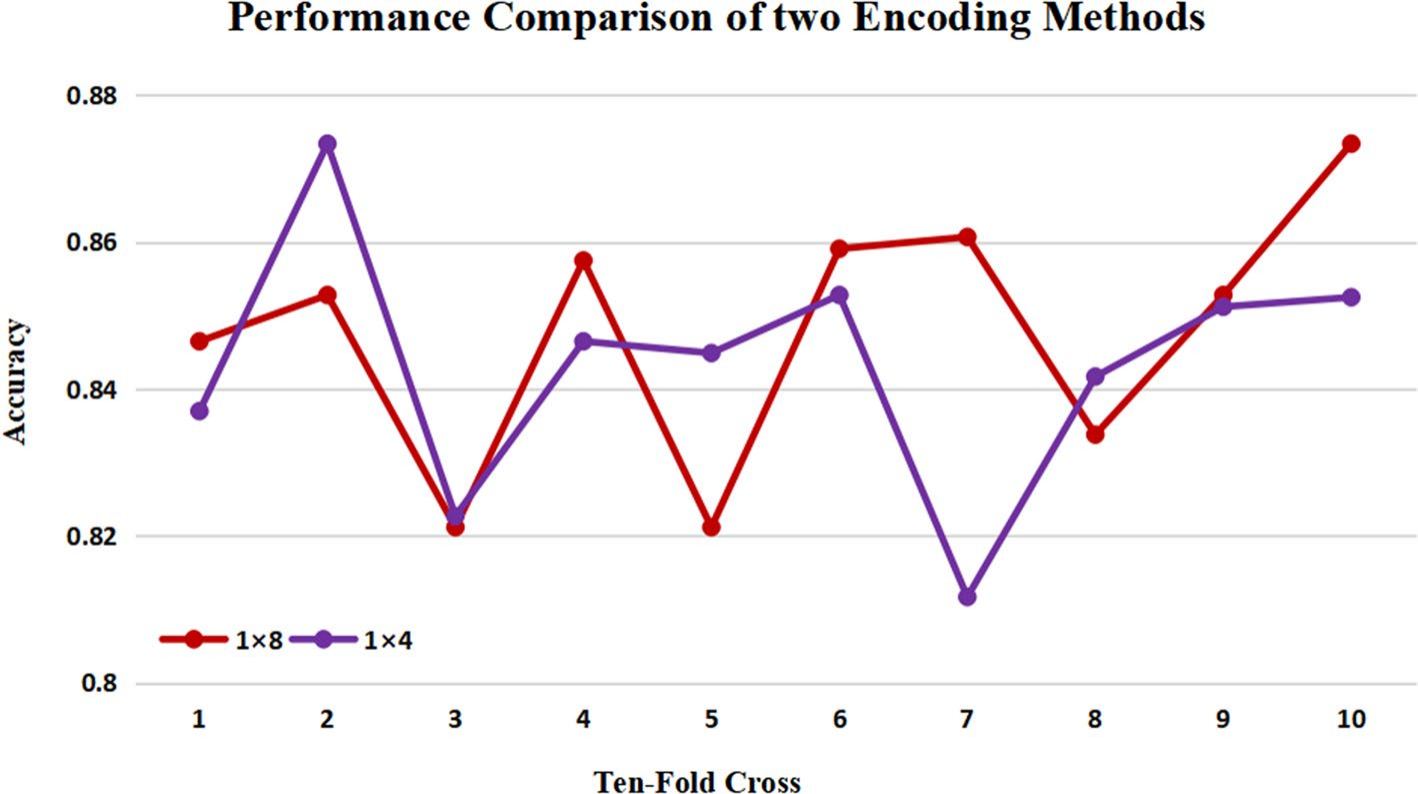
Performance comparison of each fold data in different encoding methods. The red and violet curves are the accuracy of 1×8 and 1×4 encoding methods in different fold data respectively

The data employed in this paper comes from two recent pieces of literature [18, 21], which is collected from the Rfam database [23]. It contains microRNAs, 5S_rRNA, 5.8S_rRNA, ribozymes, CD-BOX, HACA-BOX, scaRNA, tRNA, Intron_GpI, Intron_GpII, IRES, leader, and riboswitch 13 different families of non-redundant ncRNA sequences. In this paper, ten-fold cross-validation is used to test the performance of ncDLRES. The ncRNA sequences of each family are divided into ten equal parts. Among them, one part is randomly selected from each family as the test set and the remaining parts as the train set. In this way, all ncRNA sequences fall into ten-fold train and test sets. In order to facilitate ncRNA sequences input into ncDLRES, ncDLRES encodes each base into a vector. ncDLRES adopts 1×8 and 1×4 methods [21] to encode bases and selects the better one as the final encoding method. Table 2 is the conversion rule between bases and codes. A (adenine), U (uracil), G (guanine), and C (cytosine) are four common base encoding rules, while “N” represents some rare bases. Figure 4 displays the performance comparison in each fold of data under different encoding methods. Then, it can be found that in 60% of ten-fold cross, the accuracy of 1×8 is higher than that of 1×4, and the average accuracy of 1×8 is also higher than that of 1×4. Therefore, ncDLRES selects the 1×8 encoding method to encode each ncRNA sequence as L×8 (L is the length of ncRNA sequences) matrixes.

**Table 2:** The conversion rules between bases and codes

Base	A	U	C	G	N
1×8	10000010	00101000	00010100	01000001	00000000
1×4	1000	0010	0001	0100	0000

### Method

In the static deep learning model, the input data should have the same format [21]. It is necessary to pad or truncate the input data, which makes the input noise increase or features loss. Different from the static model, ncDLRES is a novel dynamic deep learning model, which directly takes ncRNA sequences with different lengths as input, thus further maintaining the integrity of the input data and making the features extracted by the method more completely. ncDLRES consists of three parts: Dynamic LSTM [25], Attention Mechanism [27], and ResNet [26]. The Dynamic LSTM can record the context information of ncRNAs with different lengths and encode them, so it is selected to extract complete ncRNAs sequence features and output the same format data. The Attention Mechanism can focus the neural network on the important features of the input data, so it is selected to focus the method on the important segments of ncRNAs sequence. ResNet can avoid the common gradient disappearance and explosion problems in the neural network, which is easy to train and has excellent performance. Therefore, it is selected to classify the output of the other two parts.

*Dynamic LSTM and Attention Mechanism:* Recurrent neural network is a kind of artificial neural network, which can record context information. Its neurons are connected according to the time sequence and can process variable-length input data. As ncRNA sequences are context sensitive text sequences, the recurrent neural network is the best network when concerning processing ncRNA sequences. Due to the limited storage space, the traditional recurrent neural network cannot effectively record the long-distance dependent information. As the length of input data increases, the traditional recurrent neural network loses its learning ability because it cannot record the feature information in an effective way. LSTM is a kind of special recurrent neural network, which can effectively solve the problem of text long-distance dependence through the special gate mechanism. LSTM contains three gates: input gate, forget gate, and output gate. To be specific, the input gate determines which information is recorded to update the LSTM hidden state. The forget gate is used to find out which useless information should be discarded at each step, while the output gate identifies output information based on the LSTM state. Furthermore, LSTM can learn long-distance dependence information at a low cost when those three gates are combined efficiently. LSTM can be performed by the following formulas (Eqs. 7–11):

7 $$\begin{aligned} i_t = \sigma (W_{xi} x_t + W_{hi}h_{t-1}+W_{ci}c_{t-1}+b_i) \end{aligned}$$

8 $$\begin{aligned} f_t = \sigma (W_{xf}x_t + W_{hf}h_{t-1}+W_{cf}c_{t-1}+b_f) \end{aligned}$$

9 $$\begin{aligned}c_t = f_t\odot c_{t-1}+i_t\odot tanh (W_{xc}x_t + W_{hc}h_{t-1} + b_c) \end{aligned}$$

10 $$\begin{aligned}o_t = \sigma (W_{xo}x_t + W_{ho}h_{t-1}+W_{co}c_{t}+b_o) \end{aligned}$$

11 $$\begin{aligned}h_t = o_t\odot tanh (c_{t}) \end{aligned}$$

where $\sigma$ is the logistic sigmoid function, while i, f, o, and c are the input gate, forget gate, output gate, and cell vector, respectively, and all of them are at the same dimension as the hidden vector h. Meanwhile, w denotes the weight matrices and b indicates the bias vectors. Equation  (7) is the calculation formula of input gate, Eq.  (8) is the calculation formula of forget gate, Eq.  (9) is the calculation formula of cell state, Eq.  (10) is the calculation formula of output gate, Eq.  (11) is the calculation formula of hidden state.

**Fig. 5:**
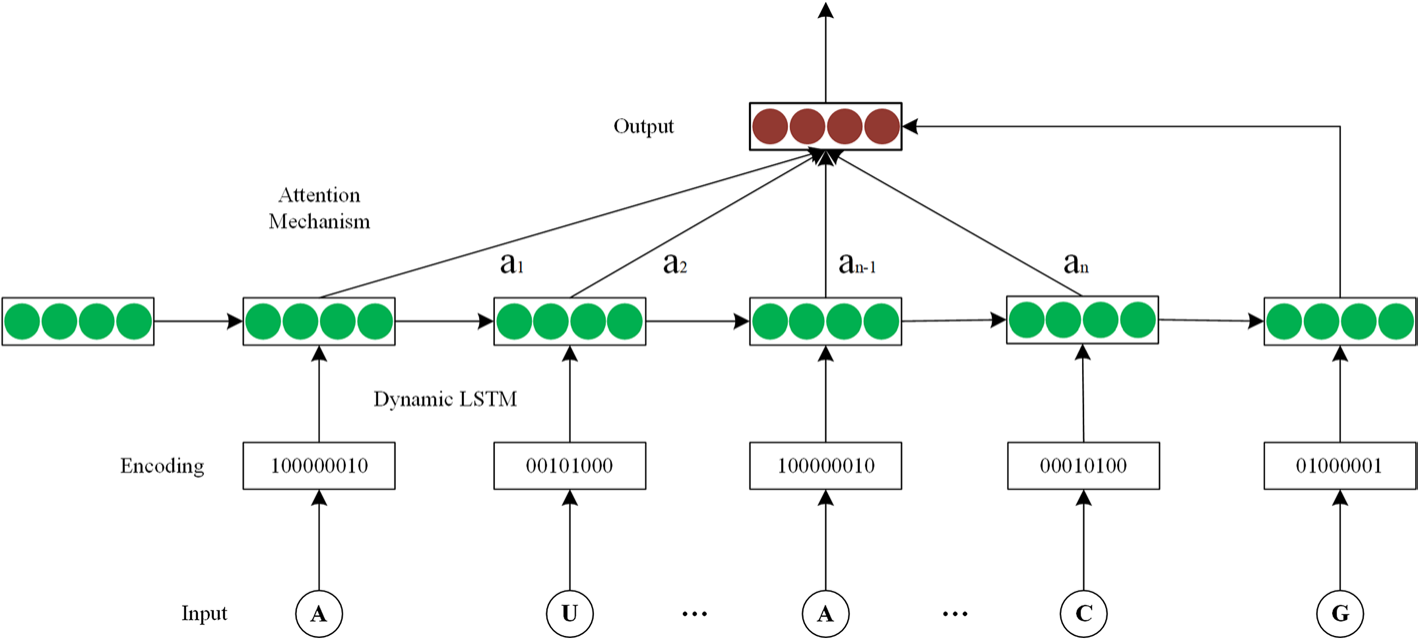
The schematic diagram of dynamic LSTM and attention mechanism. The sequences with different lengths are encoded into matrixes as the input of Dynamic LSTM. Attention Mechanism integrates the hidden state of important segments into the output with the same format

Due to the diversity of ncRNA sequence length, two methods are usually used to preprocess the data when the static deep learning model is adopted to process the data. One is padding all the sequences according to the maximum length, which not only increases the running time of the method, but also reduces the accuracy because of adding noise to the data, while the other is to intercept all the sequences into the same length sequences, which will cause the loss of sequence features and affect the prediction accuracy. Therefore, the static model cannot solve the problem of ncRNAs family prediction in the most efficient way. In this paper, one-layer Dynamic LSTM is used to solve the problem of sequence diversity. In Dynamic LSTM, all ncRNA sequences are input into the model with their real length, so that their features can be completely extracted and learned, thus improving the accuracy of family prediction. Besides, each base generates a hidden state containing context information, which is the output data of Dynamic LSTM. The same family of ncRNAs will have similar key segments. If the method pays more attention to these important segments, it can predict the ncRNAs family more effectively. The attention mechanism proposed by imitating the attention mode of human brains can complete this task in an efficient way. Attention mechanism is not a fixed neural network structure, but by adjusting the weight of attention to increase the weight of effective information, weakens the weight of invalid information. In this paper, attention mechanism is employed in ncDLRES. By learning the output of Dynamic LSTM, ncDLRES is focused on ncRNAs family segments. Figure 5 is the schematic diagram of Dynamic LSTM and Attention Mechanism.

*ResNet:* ResNet [26] is a special form of deep convolution neural network. Deep convolutional neural networks [29] have led to a series of breakthroughs, especially in the recognition and classification of two-dimensional data. Researchers have found that the number of layers is vital importance for the deep convolutional neural network [30], which can help to enrich the feature and improve accuracy. Convolution neural network will gradually reach saturation when the neurons increasing and it will have the highest accuracy in the saturation station. Hence, the accuracy of the shallow convolutional neural network will increase with the increase of depth before reaching saturation and decrease with the increase of depth after reaching saturation. When a neural network in the saturation station, if you want to increase the depth of the network and maintain the highest accuracy, the newly added layers must be the identity mapping layers, or in other words, the network needs to learn H (x) = x. In the backpropagation, the gradient will vanish or explode with the increase of network layers. Therefore, it is difficult to complete identity mapping learning. Hence, simply improving the depth of the neural network cannot meet the requirement of performance improvement. He [26] proposed ResNet in 2015 to solve the problem of neural network degradation. ResNet contains many residual blocks, which are composed of two layers of convolution neural network. Unlike the traditional convolution neural network, the ResNet uses shortcut connection to connect the input layer and the output layer, so that the mapping output of the residual block is H (x) = F (x) + x. In the residual block, the input data x is not only the input of the input layer, but also combined with the mapping of the output layer to form the output of the residual block. Experiments have proved that the newly added layer needs to learn F (x) = 0 after the ResNet network reaches saturation, which is much simpler than the traditional convolutional layer.

**Fig. 6:**
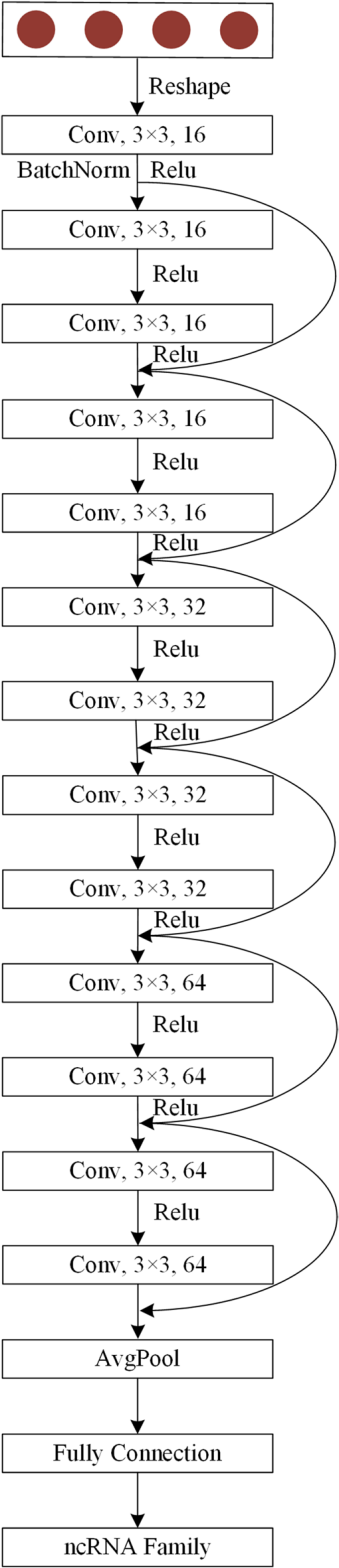
The schematic diagram of ResNet. Conv is the convolutional neural network layer; Relu is the activation function, and AvgPool is the global pooling layer

In this paper, a new ResNet that contains three types of residual blocks according to the dimension of the convolution kernel is designed and adopted in ncDLRES. As for those residual blocks, 3×3 convolution kernels are adopted, and their dimensions are 16, 32, and 64, respectively. Since the ResNet is suitable for processing two-dimensional data, the output of Dynamic LSTM and Attention Mechanism is first transformed into a matrix as the input of the ResNet. In the network of ResNet, similar to the existing ResNet, a convolution layer is used to process the input data. After that, six residual blocks are adopted to the network, which dimensions are 16, 16, 32, 32, 64, and 64 respectively. After residual blocks, the output data is 64-dimension data. Then, a global average pooling layer is used to pool the output data into 1×64 vectors. In the last, a fully connected layer is employed to classify the pooled data into the ncRNAs family. Figure 6 is the schematic diagram of ResNet.

## Data Availability

All the original experimental data can be available from the citations, and the ncDLRES model can be available at https://github.com/linyuwangPHD/ncDLRES.
