# Correction to: ncDLRES: a novel method for non‑coding RNAs family prediction based on dynamic LSTM and ResNet

**DOI:** 10.1186/s12859-021-04495-9

**Published:** 2021-12-07

**Authors:** Linyu Wang, Xiaodan Zhong, Shuo Wang, Yuanning Liu

**Affiliations:** College of Computer Science and Technology, Jilin University, Changchun, China

## Correction to: BMC Bioinformatics (2021) 22:447 10.1186/s12859-021-04365-4

Following the publication of the original article [1], the authors identified that the corresponding author was incorrectly assigned.

The incorrect corresponding author is: Linyu Wang.

The correct corresponding author is: Yuanning Liu.

The original article [1] has been corrected.

## References

[1] Wang (2021). ncDLRES: a novel method for non‑coding RNAs family prediction based on dynamic LSTM and ResNet. BMC Bioinform.

